# The introduction of an Inter-professional learning unit: staff and student perspectives

**DOI:** 10.1186/1757-1146-8-S2-P5

**Published:** 2015-09-22

**Authors:** Ainslie Davies, Jo Stephens, Michele Clark

**Affiliations:** 1 School of Clinical Sciences, Queensland University of Technology, Kelvin Grove, Qld, 4059, Australia

**Keywords:** Inter-professional learning, professional socialisation, podiatry students

## Background

The School of Clinical Sciences comprises a number of health disciplines including podiatry, paramedic science, pharmacy, medical imaging and radiation therapy. A new inter-professional unit was introduced in 2014, which covered key introductory learnings applicable for future health practitioners. This study examined teaching staff and student perspectives about their experience with the new unit for first year students.

## Methods

Qualitative interviews with teaching staff (n=9) and focus group interviews with students (5 groups which ranged in size from 4-30) were conducted. Extensive notes were taken during the interviews Issues emerging from the interviews were identified and organised according to themes and subthemes.

## Results

Four major themes were identified namely: Something new; To be or not to be that is the question; Advantages of the new unit; and Areas for improvement. Previous staff experience with inter-professional learning (IPL) had been ad-hoc, whereas the new unit brought together several disciplines in a planned and deliberate way. There was strong philosophical agreement about the value of IPL but some debate about the extent to which the unit provided IPL experience. The unit was seen as assisting students’ social and academic adjustment to university and provided opportunity for professional socialisation, exposure to macro and micro aspects of the Australian health care system and various types of communication. For podiatry students it was their first opportunity to formally meet and work with other podiatry students and moved their identity from ‘university student’ to ‘podiatry student’. Other positives included providing the opportunity for staff and students to interact at an early stage with the perceived benefit of reducing attrition. Areas for unit improvement included institutional arrangements, unit administration aspects and assessment.

## Conclusion

The unit was seen as beneficial by staff and students however, students were more polarised in their views than staff. There was a tension between feeling apart of and learning about one's own profession and feeling apart of and learning about the roles of other health professionals in relation to patient care and the health care system.

